# Closing Editorial: Electrostatics in Cell Membranes and in Artificial Membrane Models

**DOI:** 10.3390/membranes16060209

**Published:** 2026-06-11

**Authors:** Natalia Wilke

**Affiliations:** 1 Departamento de Química Biológica Ranwel Caputto, Facultad de Ciencias Químicas, Universidad Nacional de Córdoba, Córdoba X5000HUA, Argentina; natalia.wilke@unc.edu.ar; Tel.: +54-351-535-3855; 2 Centro de Investigaciones en Química Biológica de Córdoba (CIQUIBIC), Universidad Nacional de Córdoba, Córdoba X5000HUA, Argentina

## 1. Overview of the Topic

Beyond acting as structural boundaries, biological membranes function as sensitive transducers, defined by a nanometer-scale bilayer matrix embedded with proteins. The abrupt dielectric constant shift between the polar interface and the nonpolar core produces a high interfacial electric gradient, which varies due to temporal and spatial fluctuations in membrane composition. This complex electrostatic environment intimately couples with membrane mechanics to influence molecular docking and transmembrane transport. This collection brings together key contributions—spanning experimental measurements, theoretical frameworks, and computational modeling—to explore how electrostatics govern cell membrane functionality and the transduction processes occurring in both biological and artificial systems.

## 2. Summary of the Contributions to This Special Issue

The articles published in this Special Issue explore various aspects of electrostatics, mechanical properties, and ionic behavior within biological and artificial membranes. Theoretical frameworks are employed to analyze how discrete charge distributions and channel geometry influence free energy and cation selectivity. Experimental and computational studies demonstrate that membrane electrostatics—including surface, dipole, and boundary potentials—critically modulate the adsorption of pH-sensitive and cell-penetrating peptides as well as other pharmacological drugs. The role of specific lipids in structural regulation is emphasized. This Special Issue collects both theoretical/computational approaches and experimental approaches.

Within the theoretical articles, Bossa and May (Contributions 1) investigated the Debye–Hückel free energy of electric double layers in the presence of discrete charges at a dielectric interface, and found that the discrete nature of the charges becomes critical at low density charges, where dipolar interactions dominate, arising from the dipoles formed by the surface charges and their associated counterions. Zhang (Contributions 2) focused on understanding the physical parameters controlling selectivity of membrane channels to a specific ion. He studied the ionic flow through a channel with permanent charges containing two cations and one anion, as determined by the spatial distribution of acid and basic side chains of the protein. These permanent charges add an additional component to the rich behavior of these channels. Villanelo et al. (Contributions 3) also studied the effects of charges within the channels on ion flow; specifically, they analyzed the presence of the calcium ion within the connexin hemichannels. Using molecular dynamics simulations, they demonstrated that the pure electrostatic potential elicited by the full binding of calcium ions is not sufficient to block the ionic currents or the water flux. These works highlight the importance of the discrete charge distribution at short length scales, a factor that governs the fine-tuned selectivity of the processes in living systems, but is not considered in continuous models, and is usually neglected in first descriptions of a system.

Also using molecular dynamics simulations, Ruiz-Fernandez et al. (Contributions 4) explored the effect of membrane composition in ion channel gating induced by nanosecond pulsed electric fields. They showed that at high cholesterol content, the voltage-sensing domain of a voltage-gated Ca^2+^ channel undergoes major conformational changes, supporting the notion that membrane composition may act as an allosteric regulator. Non-specific effects on the lipid bilayer physicochemical properties were also observed by Zakharova et al. (Contributions 5) using several experimental methods. These authors found that phosphodiesterase type 5 inhibitors affect the transmembrane distribution of electrostatic potentials and lipid packing, which in turn leads to an enhancement in the activity of pore-forming antimicrobial agents. In turn, Choe (Contributions 6) studied membrane transport through the direct translocation of molecules with the aid of cell-penetrating peptides. He implemented a weighted ensemble method in a molecular dynamics simulation and showed that this method can be helpful in revealing information regarding the transport mechanisms of cell-penetrating peptides through various model membranes.

Aside from the presence of ions and ion gradients, local pH is another important regulator of electrostatic-driven membrane processes. This topic has been explored in articles from the collection. Alvares et al. (Contributions 7) studied how media acidity modulates peptide adsorption to anionic bilayers. Using fluorescence spectroscopy and constant pH molecular dynamics simulations, they demonstrated a high sensitivity to pH for a histidin-rich peptide with antimicrobial properties, which shows ten times higher affinity for the membrane in acidic conditions compared to neutral ones. Also related to pH changes, Fillafer et al. (Contributions 8) showed that the hydrolysis of the neurotransmitter acetylcholine by acetylcholinesterase, generating choline and acetic acid, leads to media acidification, which in turn induces a strong response in membranes composed of phosphatidylserine and phosphatidic acid, both lipids that are present in postsynaptic membranes. They proposed that cholinergic transmission is due to postsynaptic membrane protonation, an idea that has not yet been refuted. A possible role of the receptor protein would be to recruit proton-sensitive lipids, which could be excited by hydrolysis of micromolar amounts of acetylcholine.

Proton gradients have also been identified as key parameters in mitochondrial architecture (Contributions 9). Energy production occurs in specific dynamic membrane invaginations in the inner mitochondrial membrane called cristae, and the integrity of these structures is recognized as a key point for proper mitochondrial function. However, the driving forces that originate and maintain these structures are not yet fully understood. The review by Joubert and Puff examines the fundamental relationship between the architecture of mitochondrial cristae and their specific lipid composition, with a particular focus on the role of cardiolipin. It discusses how cardiolipin acts as a sensor for protons and calcium, facilitating the formation of cristae-like invaginations and optimizing the efficiency of oxidative phosphorylation by acting as a “proton trap”.

Finally, in (Contributions 10), Galassi and I discuss the intricate coupling between mechanical properties and electrostatics in biological membranes. We first describe the various electrostatic potentials that are relevant in living systems, their origin, and characteristics. Despite this being textbook information, confusion regarding the different electrostatic potentials at interfaces is frequently observed in the literature. Then, we review what has been studied about flexoelectricity in biomembranes and how these electromechanical couplings serve to partially explain signal propagation in nerve cells.

## 3. Future Perspective in Biomembrane Electrostatics

Biological membranes are highly sensitive and dynamic transducers. Structurally, they are conceptualized as a nanometer-scale, bilayer lipid matrix embedded with functional proteins. A defining feature is the sharp dielectric gradient across the membrane: the apolar lipid core (dielectric constant Ɛ ≃ 2) contrasts sharply with the surrounding aqueous phase (Ɛ ≃ 80). This dramatic change, combined with the lateral heterogeneity and dynamic, non-static composition of the membrane plane, renders interfacial electrostatics exceptionally complex, with local charge and properties being extremely important. The works in this collection represent an advance in the understanding of how discrete charge distributions define specific interactions in biology. These details, which are specific to each system of charges, are lost in continuous models and need more attention for a complete understanding of specificity in membrane channels and in protein–receptor interactions. Considering the effect of static charge distribution geometries is a starting point, but the intrinsic dynamic behavior of living systems remains less explored.

Furthermore, local electrostatics are intrinsically linked to membrane mechanical properties and composition, creating a complex interdependence. These electrostatic interactions dictate critical processes, including the binding of soluble species and molecular motion within and through the bilayer. This collection also points to the relevance of lipid composition, mechanical properties, and electrostatics of cell membranes as an active component of biological processes. Several biological processes are explained by considering two components, a specific ligand–receptor interaction in a rigid scheme isolated from the membrane. However, they can also be explained by considering emergent properties, with membranes acting as a responsible interface composed of a smart material that responds to the stimuli from the environment. This complex panorama has been largely explored; however, given its level of complexity, there is still much work to do. In addition, resistance to these proposals is usually found in the scientific community, and the dogmatic models persist as the unique mainstream explanation.

Finally, the discovery of liquid-liquid coexistence within the cell has led to a new field of study in which membrane electrostatics are affected by the presence of biocondensates, which in turn regulate the interaction between the biocondensate and the membrane. These new discoveries raise questions that are only now beginning to be answered.

